# Inulin increases the beneficial effects of rhubarb supplementation on high-fat high-sugar diet-induced metabolic disorders in mice: impact on energy expenditure, brown adipose tissue activity, and microbiota

**DOI:** 10.1080/19490976.2023.2178796

**Published:** 2023-02-20

**Authors:** Marion Régnier, Matthias Van Hul, Martin Roumain, Adrien Paquot, Alice de Wouters d’Oplinter, Francesco Suriano, Amandine Everard, Nathalie M. Delzenne, Giulio G. Muccioli, Patrice D. Cani

**Affiliations:** a Metabolism and Nutrition Research Group, Louvain Drug Research Institute, UCLouvain, Université catholique de Louvain, Brussels, Belgium; b WELBIO asbl, Walloon Excellence in Life Sciences and BIOtechnology (WELBIO), Wavre, Belgium; c Bioanalysis and Pharmacology of Bioactive Lipids Research Group (BPBL), Louvain Drug Research Institute (LDRI), UCLouvain, Université catholique de Louvain, Brussels, Belgium; d current address: Institute of Biomedicine, Department of Medical Biochemistry and Cell Biology, University of Gothenburg, Gothenburg, Sweden

**Keywords:** obesity, high-fat diet high-sucrose, obesity, prebiotics, brown adipose tissue, energy expenditure, inulin, rhubarb extract, gut microbiota

## Abstract

Consumption of prebiotics and plant-based compounds have many beneficial health effects through modulation of gut microbiota composition and are considered as promising nutritional strategy for the treatment of metabolic diseases. In the present study, we assessed the separated and combined effects of inulin and rhubarb on diet-induced metabolic disease in mice. We showed that supplementation with both inulin and rhubarb abolished the total body and fat mass gain upon high-fat and high-sucrose diet (HFHS) as well as several obesity-associated metabolic disorders. These effects were associated with increased energy expenditure, lower whitening of the brown adipose tissue, higher mitochondria activity and increased expression of lipolytic markers in white adipose tissue. Despite modifications of intestinal gut microbiota and bile acid compositions by inulin or rhubarb alone, combination of both inulin and rhubarb had minor additional impact on these parameters. However, the combination of inulin and rhubarb increased the expression of several antimicrobial peptides and higher goblet cell numbers, thereby suggesting a reinforcement of the gut barrier. Together, these results suggest that the combination of inulin and rhubarb in mice potentiates beneficial effects of separated rhubarb and inulin on HFHS-related metabolic disease and could be considered as nutritional strategy for the prevention and treatment of obesity and related pathologies.

## Introduction

The prevalence of obesity continues to increase in Westernized countries^1,2^ and is associated with raising rates of obesity-related metabolic diseases such as metabolic syndrome, type 2 diabetes (T2D), cardiovascular diseases, and kidney diseases.^3,4^ According to the alarming predictions of the World Health Organization (WHO), almost one billion adults are estimated to be obese by 2025.^5^ Considering the burden of obesity worldwide and its consequences on public health, there is a compelling need to intensify efforts in order to identify new pharmacological and non-pharmacological therapies that present beneficial properties on obesity-related disorders. This could lead to the implementation of effective interventions that would help slowdown the projected increase of obesity prevalence in the coming years.

Obesity is multifactorial and involves genetical, hormonal, and environmental factors. Its etiology is strongly associated with the imbalance between energy consumed and energy expended.^6,7^ As a result, obesity is metabolically defined by excessive and abnormal energy storage as fat in white adipose tissues. Energy overload chronically leads to ectopic fat accumulation in liver and muscles, which participates to the pathogenesis of hepatic steatosis, insulin resistance, and T2D.^8,9^ By contrast, brown adipose tissue (BAT) has thermogenic capacities since it is responsible for transforming energy into heat.^10,11^ Studies in animals have shown that low-grade inflammation^12^ and increased levels of lipopolysaccharides (LPS) termed as metabolic endotoxemia^13^ that both occur in obesity, lead to alterations in dialog between metabolic organs including gut, adipose tissue, liver, and muscles, thereby resulting in ectopic fat accumulation and insulin resistance.^14,15^ Gut-derived metabolites such as secondary bile acids (BA),^16^ short-chain fatty acids (SCFA),^17^ tryptophan metabolites,^18^ amino acids,^19,20^ polyamines,^21^ and sphingolipids^22^ act as important mediators in this dialog by influencing host physiology and triggering responses at local and peripheral levels. Notably, SCFA, including acetate, propionate, and butyrate, are used as an energy source to the host and play an important role as metabolic regulators by modulating brown adipose tissue activation,^23^ liver mitochondrial function,^24^ insulin secretion by β-pancreatic cells and whole-body energy homeostasis.^25^

Diet and edible part of plants or their extracts including fibers and polyphenols are key factors in determining gut microbiota composition and gut-derived metabolites profile.^26–30^ The so-called prebiotics can be used by specific bacterial species such as *Bifidobacterium* spp., and thereby confer beneficial effects to the host.^31^ Prebiotics include inulin-type fructans (ITF). These non-digestible dietary fibers promote the growth of specific bacteria such as *Bifidobacterium* spp.^32,33^ Preclinical and clinical studies have demonstrated that administration of ITF improved obesity-induced adiposity, lipid metabolism, glucose homeostasis, and insulin resistance, by a mechanism involving gut peptides.^32,34–41^ Rheum palmatum-derived rhubarb extract is an anthraquinone-rich crude extract from rhubarb roots known to have antioxidant, anti-cancer, and anti-inflammatory effects.^42–50^ We have previously demonstrated that supplementation with rhubarb extract prevents diet-induced obesity, hepatic steatosis, adiposity, diabetes, and inflammation, and is associated with an increase in the abundance of *Akkermansia muciniphila*.^41^

Considering the importance of diet-induced modulation of the gut microbiota composition in the onset of obesity-related disorders and in particular the beneficial role of ITF and Rheum palmatum-derived rhubarb extract, the aim of this study was to investigate the effects of rhubarb and ITF, alone or in combination, on gut microbiota composition and development of obesity-related disorders in mice fed a high-fat and high-sucrose diet (HFHS). For this purpose, we performed in-depth metabolic phenotyping using technologies such as metabolic chambers, to precisely address the effects of rhubarb and/or inulin supplementation on energy metabolism. As we previously showed, we confirm that rhubarb supplementation improved diet-induced obesity, adiposity, and inflammation. More interestingly, we highlight a stronger effect of rhubarb when combined with inulin on these parameters and make the link with higher energy expenditure, lipolysis, and brown adipose tissue activity. These effects are associated with changes in gut microbiota composition, bile acids, and SCFA profiles. Our results demonstrate the beneficial effect of the association of two dietary supplements, inulin and rhubarb, on diet-induced obesity in mice and illustrate the potential of combining two dietary supplements as an efficient dietary intervention to attenuate the development of obesity.

## Results

### Rhubarb alone, or in combination with inulin improved diet-induced obesity

**Figure 1.**
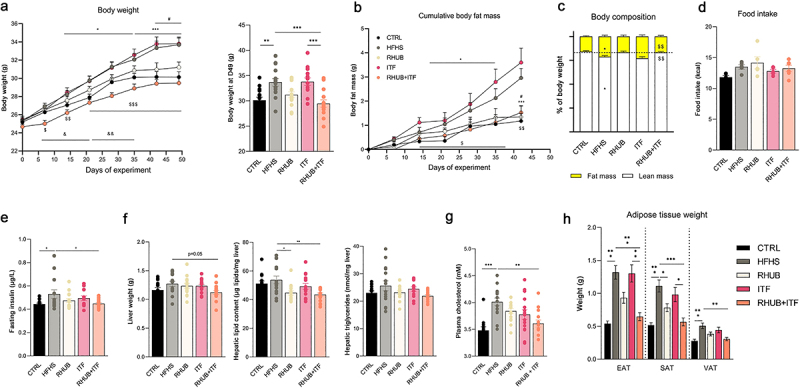
Rhubarb and inulin improve body weight gain and adiposity in response to high-fat and high-sucrose diet (a) Body weight (g) over a 7 weeks period and final body weight. (b) Cumulative fat mass gain (g) from day 0 to day 42. (c) Body composition (fat mass and lean mass) as a percentage of total body weight at the end of the experiment. (d) Mean food intake (kcal) over the 7 weeks period. (e) Plasmatic insulin levels after 6 h of fasting (µg/L). (f) Liver weight (g), total hepatic lipid content (µg lipids/mg liver) and hepatic triglycerides (nmol/mg liver). (g) Total plasma cholesterol (mM). (h) Adipose tissue weights (mg) measured after 7 weeks of experiment. EAT, epididymal adipose tissue, SAT, subcutaneous adipose tissue, VAT, visceral adipose tissue. Black, CTRL fed mice; Red, HFHS fed mice; Green, Rhubarb-supplemented mice (RHUB); Blue, Inulin-supplemented mice (ITF); Yellow, mice supplemented with both rhubarb and inulin (RHUB+ITF). Data represent mean ± S.E.M. For panel A, B and C, * represents a significant difference between CTRL and HFHS groups; # represents a significant difference between HFHS and RHUB groups; $ represents a significant difference between ITF and RHUB+ITF; & represents a significant difference between RHUB and RHUB+ITF groups. For all other panel, * represents significant difference between indicated groups. *^,#,$,&^ p ≤ .05, **^,##,$$,&&^ p ≤ .01, ***^,###,$$$,&&&^ p ≤ .001. n = 15/group.

First, we assessed the metabolic phenotype of the different groups during 7 weeks, starting at the age of 8 weeks. Compared to the control (CTRL) group, mice fed a HFHS diet gained significantly more weight already after 2 weeks and remained heavier throughout the experiment (Figure 1a). Mice fed a HFHS supplemented with rhubarb, but not inulin gained significantly less weight compared to HFHS fed mice and their body weight was comparable to those of CTRL mice (Figure 1a). Despite the lack of effect of inulin alone on body weight, mice fed an HFHS diet and exposed to both rhubarb and inulin exhibited even lower body weight all along the experiment (does not reach the significance, p = .37) than the CTRL group (Figure 1a). In line with the results on body weight, HFHS and ITF groups had a greater fat mass and fat mass/lean mass ratio compared to the other groups (Figure 1b and 1c). Fat mass of rhubarb (RHUB) and RHUB+ITF groups remained similar to CTRL group throughout the experiment. These phenotypic observations were not related to changes in food intake since all mice fed a HFHS diet showed similar increased in food intake, independent of any supplementation (Figure 1d). Plasma levels of insulin were significantly increased in mice fed a HFHS when compared to CTRL mice, but this effect tends to be alleviated when mice were supplemented with either rhubarb (p = .37) or inulin (p = .72, Figure 1e). Combination of inulin and rhubarb resulted in a significant decrease in fasting insulin to levels similar to the CTRL group (Figure 1e). At the end of the experiment, liver weight and hepatic triglycerides content were unaffected across the different groups, although mice supplemented with both rhubarb and inulin had a lighter liver as compared to HFHS mice (p = .05, Figure 1f). However, supplementation with rhubarb, alone or in combination with inulin resulted in a significant decrease in hepatic total lipid content compared to HFHS mice. Plasmatic cholesterol levels were significantly increased in mice fed a HFHS diet compared to CTRL group, and this effect was partially blunted after supplementation with rhubarb and inulin (Figure 1g). These effects were associated with significant decrease in plasma triglycerides but no change in hepatic cholesterol (data not shown). Major changes occurred with regard to adipose tissue weight, with all fat pads being smaller in rhubarb-treated mice when compared to HFHS diet fed mice (Figure 1h). Interestingly, this effect is amplified in mice supplemented with both inulin and rhubarb despite the lack of effect of inulin on itself, suggesting a synergistic effect of inulin and rhubarb on adipose tissue biology (Figure 1h). These results confirm the beneficial effects of rhubarb on body weight and fat mass gain, and highlight a potential synergistic effect of rhubarb and inulin on these parameters.

### Rhubarb and inulin act together to increase energy expenditure without altering physical activity

**Figure 2.**
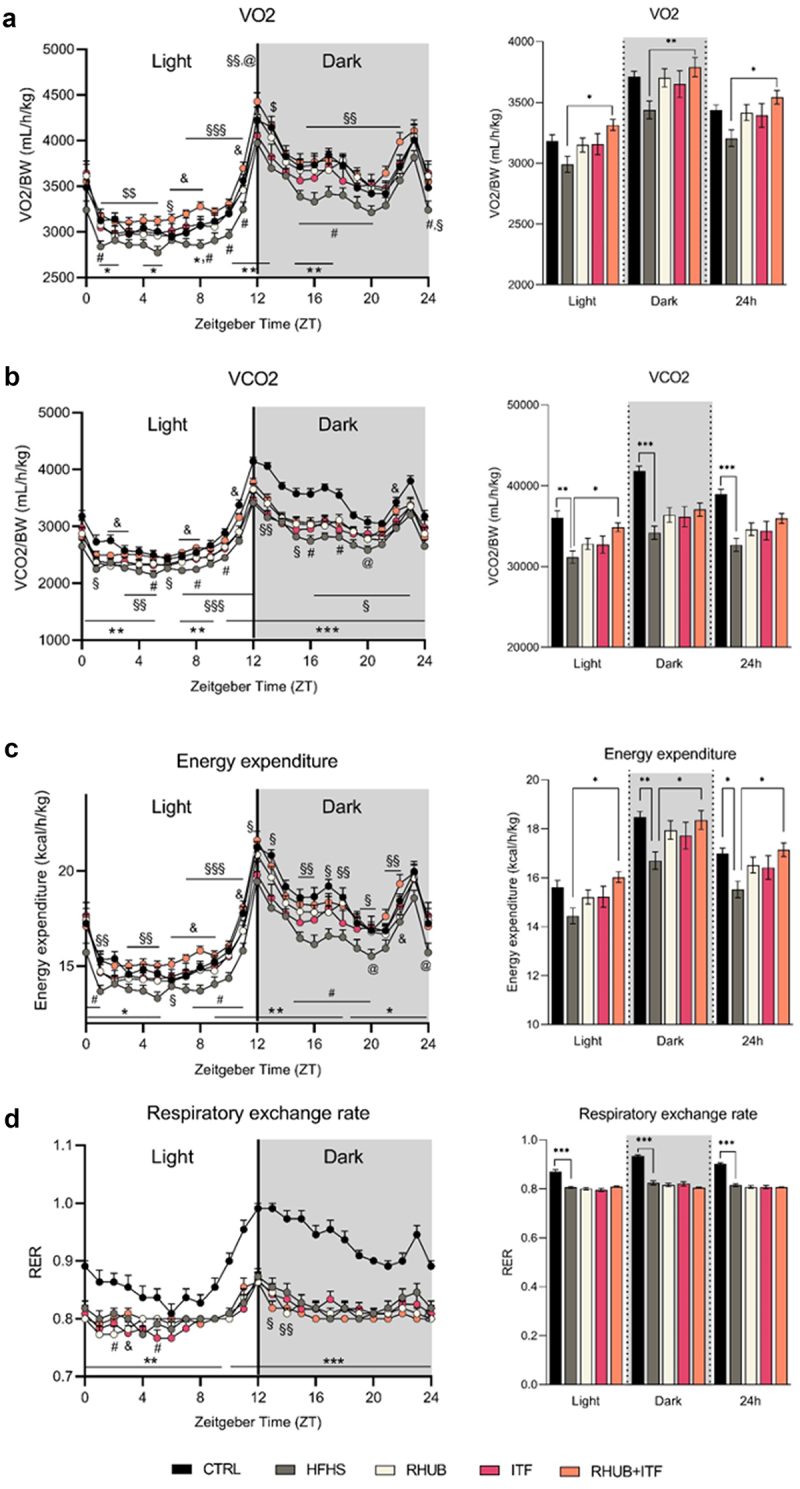
Rhubarb and inulin together increase energy expenditure and substrate oxidation (a) Left panel, daily oxygen consumption (VO2, mL/h/kg) normalized to body weight during the light phase (from ZT0 to ZT12) and dark phase (from ZT12 to ZT24) determined by indirect calorimetry analysis. Clear and shaded zones indicate light phase and dark phase, respectively. Time is expressed in Zeitgeber time and considers ZT0 as the time when light turns on and ZT12 when light turns off. Right panel, oxygen consumption normalized to body weight per phase (light, dark and whole day) per mouse (mL/h/kg). (b) Daily carbon dioxide released (VCO2, mL/h/kg) normalized to body weight. Carbon dioxide released normalized to body weight per phase. (c) Daily energy expenditure as percentage of body weight (kcal/h/kg). Energy expenditure per phase and per mouse. (d) Daily respiratory exchange ratio (RER). Data represent mean ± S.E.M. * represents a significant difference between CTRL and HFHS groups; # represents a significant difference between HFHS and RHUB groups; @ represents a significant difference between HFHS and ITF groups; $ represents a significant difference between ITF and RHUB+ITF; & represents a significant difference between RHUB and RHUB+ITF groups. § represents a difference between HFHS and RHUB+ITF groups. *^,#,$,&,@,§^ p ≤ .05, **^,##,$$,&&,@@,§§^ p ≤ .01, ***^,###,$$$,&&&,@@@,§§§^ p ≤ .001. n = 12–13/group.

To further evaluate the mechanisms involved in the protection against diet-induced obesity by rhubarb and inulin, we recorded the individual and combined effects of these dietary supplements on energy metabolism using metabolic chambers, in which mice were housed individually during the last week of the experiment and measurements were performed during the last 4 days. The results from indirect calorimetry revealed a linear decrease in oxygen consumption in HFHS group that is attenuated in mice supplemented with either rhubarb (p = .31) or inulin (p = .36) and totally abolished in RHUB+ITF group (Figure 2a). This is associated with a slight increase in VCO2 (carbon dioxide excretion) in RHUB+ITF group when compared to the HFHS group (p = .19 in dark phase and p = .058 over a 24 h period), which is significant only during the light phase (Figure 2b). The total energy expenditure was calculated from VO2, VCO2, and nitrogen excretion. The results revealed a mean decrease in energy expenditure in mice fed a HFHS diet, and this effect was partially corrected by the supplementation with either rhubarb (p = .37) or inulin (0.41) while the combination of these two compounds totally normalized the energy expenditure (Figure 2c). Measurement of physical activity (XYZ activity, ambulatory activity, and fine activity) revealed no significant differences among the groups, suggesting that changes in VO2, VCO2, and energy expenditure were unrelated to modulation in spontaneous and basal physical activity (supplementary figures 1A, B, and C). While HFHS-fed mice exhibited a lower respiratory exchange rate, which reflects lipid oxidation, no shift was observed for the supplemented groups, suggesting that neither rhubarb nor inulin affected substrate used for energy production (Figure 2d). Taken together, these results showed a global increase in energy expenditure in mice supplemented with both rhubarb and inulin, which may explain, at least partially, the protection against diet-induced obesity and fat mass accumulation.

### Rhubarb and inulin synergistically increase brown adipose tissue activity

**Figure 3.**
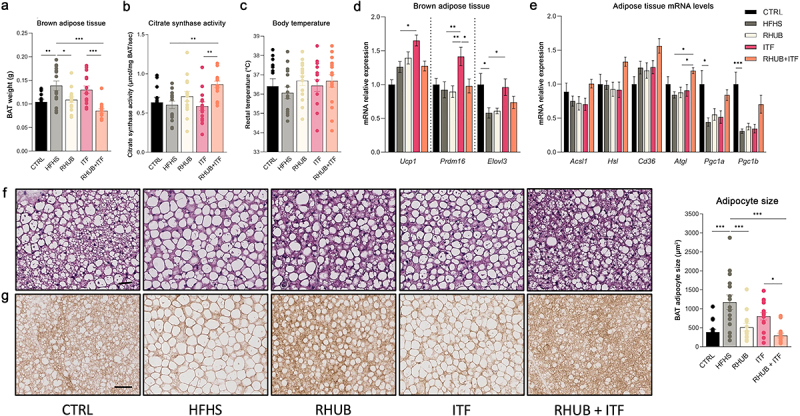
Inulin and rhubarb increase BAT activity and lipolysis (a) Brown adipose tissue weight (g). (b) Brown adipose tissue citrate synthase activity (µmol/mg BAT/sec). (c) Rectal body temperature (°C). (d) mRNA relative expression of *Ucp1, Prdm16* and *Elovl3* in BAT. (e) mRNA relative expression of *Acsl1, Hsl, Cd36, Atgl, Pgc1a, Pgc1b* in subcutaneous adipose tissue. (f) Representative pictures of brown adipose tissue Hematoxylin and Eosin staining. Scale bar, 25 µM (magnification is 20x). Adipocyte size (µm^2^) measured in 5 different sections of brown adipose tissue per mouse. (g) Representative images of staining for UCP1 in BAT. Scale bar, 25 µm (magnification is 20x). Data represent mean ± S.E.M. * represents significant difference between indicated groups. * p ≤ .05, ** p ≤ .01, *** p ≤ .001. n = 15/group.

Besides physical activity and basal metabolism, energy expenditure is also determined by the brown adipose tissue thermogenesis. To determine whether the increase in energy expenditure was associated with increased thermogenesis, we further explored the adipose tissue metabolism. First, we found that the RHUB+ITF group exhibited a large decrease in BAT weight, which was even lower than the one of the CTRL and RHUB groups (Figure 3a). This was associated with a significant increase in citrate synthase activity in mice supplemented with both rhubarb and inulin, suggesting that these compounds acted in synergy to increase mitochondrial activity (Figure 3b). The measurement of body temperature revealed no flagrant differences among the groups (Figure 3c). Then, we explored the transcriptional effects of rhubarb and inulin supplementations in the brown and white adipose tissues. While supplementation with inulin alone led to increased expressions of thermogenic markers including *Ucp1, Prdm16* and *Elovl3* in the BAT as compared to HFHS group, addition of rhubarb to the mixture abolished this effect (Figure 3d). Since adipose tissue lipolysis participates to the activation of BAT by producing free fatty acids, we measured the expression of lipolytic markers in the subcutaneous adipose tissue. The expression of *Acsl1, Hsl, Cd36, Atgl, Pgc1α* and *Pgc1b* increased when combining inulin with rhubarb as compared to HFHS group, thereby reinforcing the oxidative potential of rhubarb and inulin together (Figure 3e). To further investigate whether the increase in BAT activity involves changes in adipocyte morphology, we measured the size of adipocytes in brown adipose tissue sections. Brown fat cells from HFHS-fed mice acquired a white-like appearance, which involved a significant increase in size and a shift toward a unilocular morphology (Figure 3f). The morphology and size of adipocytes of inulin-treated mice appear similar to those of mice fed a HFHS diet. This may be linked to elevated expression of *Elovl3* in these mice (Figure 3d), which is involved in endogenous triglyceride synthesis and their accumulation into brown adipocytes. Compared to HFHS-fed mice, brown adipocytes from rhubarb-supplemented mice were smaller and displayed a more classical brown-like multilocular morphology. This effect was emphasized in the RHUB+ITF group for which the adipocyte size was even lower than that of mice fed a CTRL diet (Figure 3f). UCP1 immunohistochemistry demonstrated that the multilocular adipocytes from RHUB+ITF supplemented group exhibited a high degree of UCP1 immunoreactivity (Figure 3g). These results showed that mice supplemented with both rhubarb and inulin have an increase in BAT activity and adipose tissue lipolysis which are concomitant with the increase in energy expenditure.

### Synergy of rhubarb and inulin improves intestinal barrier function

**Figure 4.**
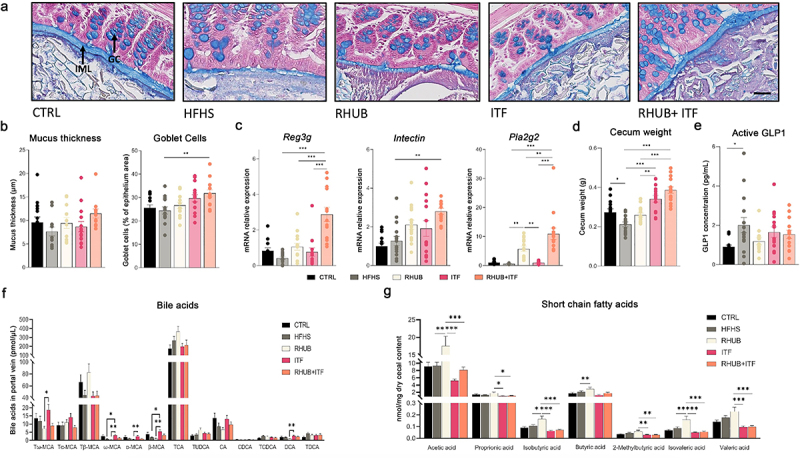
Combination of inulin and rhubarb improve gut barrier function (a) Representative pictures of colon Alcian blue staining showing inner mucus layer (IML) and goblet cells (GC). Scale bar, 25 µm (magnification is 20x). (b) Mucus thickness measured over the entire colon diameter on at least three different sections per mice. Mucus in goblet cells measured as % of blue in the epithelium in at least 3 different sections per mice. (c) mRNA relative expression of *Reg3g, Intectin* and *Pla2g2* in the colon. (d) Cecum weight (g). (e) Active GLP1 concentration (pg/mL) in vena porta. (f) Bile acid concentration of conjugated and unconjugated bile acids (pmol/µL) in vena porta. (g) Short-chain fatty acid concentration in cecal content (nmol/mg dry cecal content). Data represent mean ± S.E.M. * represents significant difference between indicated groups. * p ≤ .05, ** p ≤ .01, *** p ≤ .001. n = 15/group.

Maintenance of intestinal homeostasis involves different lines of defense in which the intestinal mucus layer is a key component since it protects against mechanical, chemical, and biological attacks.^51^ To assess mucus quality and functionality, we measured the mucus thickness (intestinal mucus layer; IML) and the goblet cells/enterocytes ratio (GC) on Carnoy fixed mouse distal colon. Histological results revealed a 20% decrease in the mucus thickness in HFHS treated mice (P > .05) and an 33% increase IML in mice fed a HFHS diet and supplemented with rhubarb and inulin, but these effects did not reach significance (p = 0,1) (Figure 4a and 4b). These mice also exhibited a significant increase of Alcian blue-positive Goblet cells in the intestinal crypts as compared to HFHS mice (Figure 4b). To further investigate the effects of inulin and rhubarb on the intestinal barrier, we measured the expression of antimicrobial factors (*Reg3g; Pla2g2*) and cell renewal marker, *Intectin*. First, we confirmed that supplementation with rhubarb leads to an increase in the expression of *Reg3g* and *Pla2g2*, and we bring evidence of a synergistic effect of rhubarb and inulin on the expression of the three markers since their expressions are even more induced in the presence of both inulin and rhubarb (Figure 4c). These results, together with the increase in the quantity of GC producing mucins highlight the synergistic effect of rhubarb and inulin as a potential modulator of the intestinal barrier.

In addition, we observed a mild increase in the cecum weight in the RHUB group and an even bigger increase in mice supplemented with inulin alone or in combination with rhubarb, probably as a result of the high microbial fermentation of inulin (Figure 4d). Due to the important microbial fermentation of rhubarb and inulin in the colon, we assessed the capacity of these compounds to modulate gut-derived glucagon-like peptide-1 (GLP1) secretion. Measurements of GLP1 concentrations in portal vein revealed no differences among the groups except a surprising increased GLP1 concentration in HFHS fed mice, suggesting that neither inulin nor rhubarb influenced GLP1 secretion in this context (Figure 4e). In line with these results, we found no differences in BA profiles in RHUB+ITF mice, suggesting that the synergistic effect of rhubarb and inulin on lipid and energy metabolism does not require a modulation of the bile acid pool (Figure 4f). Of note, the unconjugated/conjugated bile acid ratio showed a shift from conjugated to unconjugated bile acids in mice supplemented with inulin alone, suggesting that inulin alone is sufficient to influence the deconjugation process of bile acids in the intestine (Supplemental Figure 2).

Next, we measured the levels of short chain fatty acids in cecal content. These lipid species are directly produced by the gut microbiota and influence peripheral metabolism including adipose tissue metabolism. We found that rhubarb significantly increased both SCFA and branched SCFA, while inulin tended to decrease these levels (Figure 4g). Addition of rhubarb to inulin was not sufficient to normalize SCFA levels, suggesting a major effect of inulin on modulating SCFA levels.

### Specific gut microbiota profile in response to the supplementation of inulin and rhubarb

**Figure 5.**
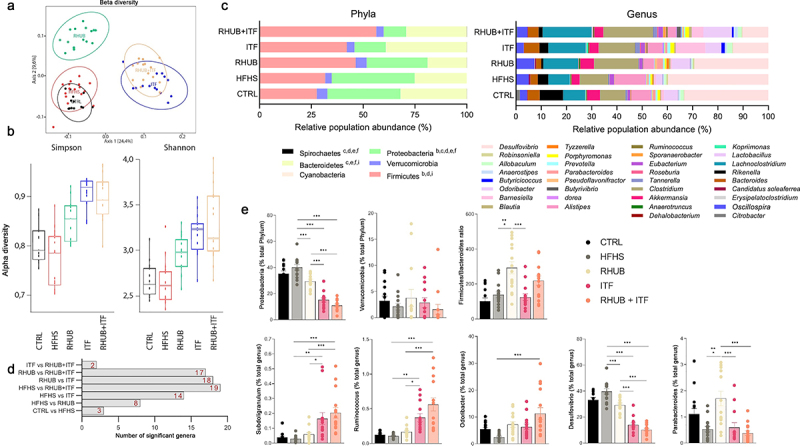
Inulin alone or in combination with rhubarb leads to a shift in the composition of gut microbiota (a) Non-metric multidimensional scaling (MDS) representing the Jaccard-binary differences between individuals within groups. (b) Simpson and Shannon alpha-diversity from cecal content. (c) Relative abundance obtained by OTUs of the major bacterial phyla (left) and genera (right). (d) Number of significant genera differentially modulated across the conditions, based on Kruskal-Wallis comparison test. (e) Relative abundance of specific bacterial phyla and genera in each sample among the groups. (c,d, e) Kruskal-Wallis with Dunn’s multiple comparison test. Data represent mean ± S.E.M. * represents significant difference between indicated groups. * p ≤ .05, ** p ≤ .01, *** p ≤ .001. n = 15/group.

By using 16S rRNA sequencing, we measured the abundance of bacterial DNA at different taxonomic levels. Multidimensional scaling plots revealed that all supplementation conditions caused a shift in microbiota composition either along the axis 1 (ITF and RHUB+ITF groups) or the axis 2 (RHUB group), explaining more than 24% and 9% of the differences, respectively (Figure 5a). This untargeted picture of beta-diversity revealed that inulin has more influence on the composition of gut microbiota than rhubarb since 24.4% of the differences are explained by the presence of inulin, independently from rhubarb. In line with results on beta-diversity, analysis of alpha-diversity by Simpson and Shannon indexes revealed a higher community heterogeneity for all the supplemented groups, where the effect of inulin alone or in combination with rhubarb was dominant (Figure 5b). At the phylum level, clear differences were observed in mice supplemented with rhubarb and/or inulin. While the abundance of Verrucomicrobia was unchanged, DNA sequencing revealed a lower abundance of Proteobacteria in RHUB and ITF mice (higher effect in ITF mice), which is accentuated when rhubarb and inulin are combined (4-fold decrease) (Figure 5c and e). Abundance of Firmicutes is higher in all groups of treated mice and Firmicutes/Bacteroidetes ratio is significantly increased in mice supplemented with rhubarb alone or in combination with inulin (Figure 5e). At the genus level, we first confirmed previous finding showing that rhubarb supplementation tends to increase the abundance of *Ruminococcus, Odoribacter* and *Parabacteroides* and decreased the abundance of *Desulfovibrio* (Figure 5e). Importantly, we observed that the changes occurring in the RHUB+ITF group were primarily due to the effects of inulin itself on genus distribution (Figure 5c, d and supplemental data). The abundance of *Subdoligranulum, Ruminococcus, Desulfovibrio* and *Parabacteroides* varied in a direction determined by the presence of inulin since the genus profile of RHUB+ITF mice mirrored that of ITF but not RHUB mice (Figure 5e). It is worth noting that the combination of rhubarb and inulin amplified the effect of inulin alone on genus distribution. Altogether, these results highlight different gut microbiota profile and composition between HFHS-fed mice and the mice supplemented with inulin, rhubarb, or a combination of both and show that there is a synergistic effect of rhubarb and inulin on the abundance of several genera.

## Discussion

Inulin and rhubarb have both been described as beneficial nutritional compounds for the host. Preclinical and clinical studies revealed that inulin prebiotic decreases adiposity, fasting glucose levels, energy intake, and inflammation, especially in the context of obesity.^35,52–56^ In addition, we previously demonstrated the beneficial metabolic effects of rhubarb in obesity, which included reduced body weight gain, hepatic steatosis, insulinemia and inflammation.^41^ The current study aimed at investigating the potential synergistic effect of inulin and plant-derived rhubarb extract in a murine model of diet-induced obesity. We demonstrated that the combination of both inulin and rhubarb abolished diet-induced body weight gain and fat mass accumulation. The use of metabolic chambers and in depth-analysis including transcriptomic, histological, and biochemical analysis allowed us to explore the molecular mechanisms involved. These effects were associated with the maintenance of energy expenditure, brown adipose tissue activity and white adipose tissue lipolysis and a remodeling of gut microbiota composition. It is important to note that inulin alone failed to decrease body weight gain and adiposity while its combination with rhubarb revealed its metabolic efficiency. We showed that rhubarb is the main driver of effects on metabolism since rhubarb supplementation alone is sufficient to decrease body weight gain, adiposity and increase brown adipose tissue activity and maintain gut barrier integrity. Interestingly, we demonstrated that these beneficial effects are even more important when rhubarb is combined to inulin, which means that the presence of inulin enhances the metabolic benefits of rhubarb in this context. These data suggest a potential synergistic mode of action that may involve several mechanisms. Do rhubarb and inulin need to physically interact to exert their effects on metabolism or does inulin create a microbial positive environment that allows the full metabolic capacities of rhubarb?

We believe the second hypothesis is the most probable since we showed that in comparison to rhubarb, inulin is highly fermented in the intestine and is responsible for significant modifications of gut microbiota composition, whether alone or in combination with rhubarb. Thus, we hypothesize that the synergistic effect of inulin and rhubarb involves a two-hit metabolic process that begins with the prebiotic effect of inulin on rhubarb metabolization and fermentation in the intestine, which secondly enhance its own beneficial properties to the host. In other words, inulin may drive changes in microbiota composition in a way that allows a better microbial transformation of rhubarb, thereby strengthening its beneficial effects on local and peripheral metabolism.

Inulin-derived fibers may exert an important role in this synergistic two-hit process. Inulin is a dietary fiber found in some fruits and vegetables, which is prone to colonic fermentation and promotes health benefits. Thus, inulin-derived fibers could increase the beneficial properties of rhubarb by creating a specific niche for the gut microbial transformation of rhubarb. The mode of action of fibers involves SCFA, which are directly derived from dietary fibers and act both locally, as a source of energy for epithelial cells, and at the periphery to modulate glucose and lipid metabolism in the liver and the adipose tissue.^57^ However, our results show that inulin supplementation, when associated with a HFHS diet, does not promote an increase in SCFA levels in cecal content, suggesting some independent mechanisms of action. Regarding rhubarb composition, it is mostly composed of anthraquinones, anthrones, stilbenes, and phenolic compounds. The combination of these chemical structures is likely responsible for the hypolipidemic effects, as already described in other models of obesity and in vitro experiments.^58,59^ Anti-obesity agents from rhubarb extract mostly target adipogenesis through CCAAT-enhancer-binding proteins (C/EBPs) and peroxisome proliferator-activated receptors (PPARs).^60,61^ Nevertheless, we cannot exclude that the benefits of rhubarb on glucose and lipid metabolism may also involve other constituents such as low-molecular phytochemicals. In this context, phytocompounds from rhubarb and inulin may exert a synergic effect and boost lipid-lowering action of each other.

Other plant-derived components may be responsible for the beneficial effects of rhubarb and inulin. Dietary flavonoids, which are widely present in plant-based food, are also known to exert beneficial effects to the host. Interestingly, they are also susceptible to be microbially transformed, a process that subsequently alters their absorption, bioavailability, and metabolic function.^62–64^

Macronutrients and plant-derived components are key determinants of the gut microbiota homeostasis. They directly or indirectly affect the physical and chemical components of the intestine including the mucus layer, tight junction proteins, immune cells, and antimicrobial factors.^26^ The present study showed that while HFHS does not alter gut microbial environment, supplementation with both rhubarb and inulin significantly increased the expressions of antimicrobial factors like *Reg3g* and *Pla2g2* and the cell-renewal protein *Intectin* and increased the quantity of Goblet cells-producing mucus, albeit without influencing mucus thickness *per se*. These results are in line with other studies showing that mucus growth rate, rather than the simple mucus thickness is a better indicator of mucus turnover process.^65,66^ Moreover, these results strengthened our previous findings showing that rhubarb supplementation alone was sufficient to increase the expressions of *Intectin, Reg3g* and *Pla2g2* and highlighted the importance of inulin in increasing rhubarb-induced transcriptomic effects when the two are combined.

The gut microbiota also produces/alters mediators including BA and SCFA that influence microbe-host dialog and their related metabolic pathways, thereby contributing to maintain metabolic homeostasis.^67,68^ Here, we showed that the combination of rhubarb and inulin does not significantly influence BA metabolism nor the plasmatic levels of GLP1, which is known to be increased by bile acids through FXR and TGR5.^69–76^ This means that the metabolic outcomes resulting from the supplementation with both inulin and rhubarb are unrelated to changes in the bile acids pool. Of note, despite not significant, inulin and rhubarb have opposite effects on the proportion of conjugated and unconjugated bile acids and this effect does not reflect the variations in GLP1 concentrations for these groups, suggesting some independent mechanisms of regulation. Indeed, conjugated bile acids such as TDCA, TCA and LCA activate TGR5, which results in GLP1 release. SCFA also act as gut-derived signaling molecules and are involved in energy metabolism by binding and activating G-protein-coupled receptors (GPR)-41 and GPR43. Of interest, SCFA are well described as modulators of energy expenditure and adipose tissue metabolism since preclinical and clinical studies showed that both acute and chronic administration of SCFA increase energy expenditure.^73–77^ Here, we showed that both SCFA and branched SCFA were increased by the rhubarb but not by inulin alone or in combination. Therefore, changes in the pool of SCFA could explain, at least in part, the increase in energy expenditure and BAT activity that we observed in mice supplemented with the rhubarb treatment only and not with inulin. This mechanism could involve connections to the brain since it has been shown that SCFA act through GPR43 in the sympathetic ganglion to stimulate sympathetic nervous system activity that further led to an increase in energy expenditure.^77^ SCFA could also act indirectly on energy expenditure by increasing brown adipose tissue activity since SCFA have been shown to activate brown adipose tissue via the sympathetic nervous system.^23^ Consistent with this hypothesis, we found that rhubarb increases BAT activity, as revealed by the decrease in BAT weight and the increase in citrate synthase activity and UCP1 staining. Given that SCFA are increased by rhubarb and decreased when rhubarb is associated to inulin, these data suggest that rhubarb is likely acting via additional pathways than those controlled by SCFA. We aforementioned the capacity of chemical components of rhubarb to directly target transcription factors such as C/EBPs and PPARγ to drive an adaptative response in adipose tissue, and this could be another mechanism of action to be investigated. Another indirect mechanism that could explain the increase in energy expenditure involves adipose tissue lipolysis which deliver substrates, including free fatty acids, that fuels BAT thermogenesis.^78,79^ In the present study, we showed that mice supplemented with both rhubarb and inulin exhibited an increase in the expression of lipolytic markers in the subcutaneous adipose tissue. Therefore, the increase in energy expenditure in mice supplemented with both rhubarb and inulin may have different origins including an increase in BAT activity, an increase in WAT lipolysis and/or a stimulation of the sympathetic nervous system, all of which may or may not dependent on SCFA content.

In addition to providing evidence of a synergistic effect of rhubarb and inulin on whole metabolism, the present study also confirmed previous findings showing the beneficial effects of rhubarb alone on body weight gain, adiposity and brown adipose tissue activity.^41^ In accordance with our previous findings, we showed that rhubarb-induced beneficial effects on metabolism are associated with a remodeling of the gut microbiota, with a decrease in Proteobacteria and Bacteroidetes phyla and an increase in *Parabacteroides* and *Erysipelatoclostridium* genera. However, the present study revealed no differences in the abundance of *Akkermansia muciniphila* in rhubarb-treated mice, suggesting that beneficial effects of rhubarb are not solely due to the increased abundance of *Akkermansia muciniphila*. We also showed that inulin alone was not sufficient to promote health benefits to the host, despite improvements in gut microbiota composition and bile acid metabolism. These results support the notion that effects of inulin are contrasted; its supplementation has previously been reported as beneficial,^80–83^ neutral^84^ or deleterious^85^ in the context of obesity, metabolic syndrome, or colitis, respectively. It is worth noting that the type of diet used in this study was different from the classical high-fat diet, which contain 60% calories from fat. Here, mice were fed a HFHS diet, which reflects more the human caloric intake in the modern Western society. The increased quantity of sucrose in the HFHS diet may explain, at least in part the absence of effect of inulin supplementation, alone. However, association of inulin with other supplements such as plant-derived rhubarb, reveals the prebiotic effect of inulin on metabolism. Thus, the interest of supplementing with inulin seems linked to the composition of the diet (i.e, macronutrient and micronutrient composition); and this needs to be taken into consideration for further clinical applications. In addition, we and others have shown the inulin type fructans may have a curative effect on different models of obesity or type 2 diabetes,^86,87^ but here we have to acknowledge that we have not pretreated the mice with HFHS and therefore we can speak about a prevention of the development metabolic disorders but not a curative effect. Whether the combination of inulin and rhubarb in other models of established diseases will be effective remain to be tested.

In conclusion, our study put forth for the first time the synergistic activity of inulin and rhubarb *in vivo*. In response to a HFHS diet, supplementation with both rhubarb and inulin amplifies some of their respective metabolic beneficial effects. In-depth analysis using metabolic chambers allowed us to highlight a preservation of energy expenditure in the presence of both rhubarb and inulin that is associated with an increased brown adipose tissue activity. When considering the burden of obesity and obesity-related disorders, these findings support the idea that supplementation with plant-derived extracts combined with prebiotics could represent an interesting nutritional strategy to fight obesity-associated comorbidities via a modulation of the gut microbiota.

## Material and methods

### Mice

Eight-week-old C57Bl6/J male mice (n = 75; Janvier, France) were housed in pairs under specific conditions, i.e., specific and opportunistic pathogen-free conditions (SOPF) and in a controlled environment (12 h daylight cycle, temperature of 22 ± 2°C) with food and water *ad libitum*. After being acclimatized during one week with a control diet (CTRL) (D12450H, Research diet) and matched according to body weight and fat mass, mice were divided into 5 distinct dietary groups (n = 15/group): (1) control group of mice fed a control diet (D12450H, Research diets) containing 10% calories from fat (CTRL group); (2) mice fed a high-fat and high-sucrose diet (HFHS diet, D12451, Research diets) containing 45% calories from fat and 35% calories from carbohydrates (HFHS group); (3) mice fed a HFHS diet supplemented with rhubarb extract (0.3% w/w in the diet, Ortis, Belgium) (RHUB group), (4) mice fed a HFHS diet supplemented with inulin, a chicory root extract (20% food intake in water, Fibruline, Cosucra, Belgium) (ITF group); (5) mice fed a HFHS supplemented with rhubarb and inulin (0.3% rhubarb in the diet and 20% inulin in the drinking water) (RHUB+ITF group). Supplementation with either rhubarb, inulin or both started concomitantly with the introduction of HFHS diet. Water containing inulin was replaced every two days and the concentration of inulin was adapted depending on food intake for each cage. Body weight, food intake and water intake were recorded every week for the duration of the experiment (6 to 9 weeks). Body composition was assessed once a week by using 7,5-MHz time-domain nuclear magnetic resonance (LF50 minispec; Bruker; Rheinstetten, Germany). All mouse experiments were approved by and performed in accordance with the guideline of the local ethics committee (the ethics Committee of the Université catholique de Louvain for Animal Experiments specifically approved this study, agreement number 2017/UCL/MD/005).

At the end of the experiment and after 3 hours of fasting, all mice were anesthetized with isoflurane (Forene, Abbott, Queenborough, Ken, UK) and blood was sampled. After exsanguination, mice were euthanized by cervical dislocation. Organs and tissues were dissected, weighted, and directly immersed in liquid nitrogen before storage at −80°C for further analysis.

### Indirect calorimetry experiments

After 6 to 8 weeks of experiment, 12 or 13 mice per group were transferred to individual metabolic chambers (Phenomaster, TSE Systems, Bad Homburg, Germany) to measure whole energy expenditure, oxygen consumption, production of carbon dioxide, respiratory exchange ratio (RER), food intake and spontaneous locomotor activity. The first three parameters were calculated after correction for body weight. Activity was assessed using an infrared light beam-based locomotion monitoring system (expressed as counts per hour) along X, Y and Z directions. Fine activity represents the grooming and scratching of the mice. Ambulatory activity is the directed ambulatory locomotion that occurs above x, y, and z directions. Inside the chambers, mice had free access to food and water. Metabolic measurements started after 3 days of acclimation and lasted 4 days, from Zeitgeber time (ZT)6 (12 pm) the first day to ZT0 (6 am) the last day with a measurement recorded every 15 minutes. For each mouse, the results are represented as the mean per hour (4 periods of 15 minutes) over a 24-h period and are expressed as the mean of the 4 days of measurement. Accordingly, the final data represents the mean values, respectively, from ZT0 to ZT12 (day), ZT12 to ZT24 (night) or ZT0 to ZT24 (24 h). After 4 days of measurement, mice were euthanized after 3 hours of fasting and tissues were sampled as described above.

### Liver lipid quantification

Liver lipids were measured after extraction according to method of Folch et al., as previously described.^88^ Briefly, approximately 100 mg of liver tissue was grinded in 2 mL of CHCl_3_-MeOH (2:1, v/v) and then homogenized using an ultrasonic homogenizer. Lipids were extracted by adding 400 µL of 0.9% NaCl solution and vigorously shaking. After centrifugation, the organic phase was collected in a new glass tube and dried under nitrogen. Glass tubes were weighted before, and after, lipid extraction, in order to estimate the total lipid content. The dried residue was solubilized in 1.5 mL isopropanol. Liver triglyceride and cholesterol levels were measured using kits coupling an enzymatic reaction with spectrophotometric detection of the reaction end-products (Diasys Diagnostic and systems, Holzheim, Germany), according to the manufacturer’s instructions.

### Citrate synthase activity assay

Citrate synthase activity in the brown adipose tissue was assayed in approximately 10 mg of brown adipose tissue lysed in 20 volumes of CelLytic MT Cell Lysis containing 1% (v/v) of protease inhibitor cocktail P8340 (Sigma, Saint-Louis, MO, USA) by bead-beating. The lysate was centrifuged two times at 10,000 g during 10 min at 4°C in order to remove the lipids and the tissue debris. Tissue extract was diluted 1:10 in a 100 mM phosphate buffer (pH 7.1) containing 10 mM 5,5′-dithiobis-(2-nitrobenzoic acid) (DTNB) and 30 mM acetyl-CoA. After the addition of 10 mM oxaloacetate, free coenzyme A produced from the condensation of acetyl-CoA and oxaloacetate was bound to DTNB, and resulting change in light absorbance detected spectrophotometrically at 412 nm was used to determine the activity of citrate synthase (µmol/mg/s).

### Plasmatic analyses

Circulating glucagon-like peptide-1 (GLP1) concentration was determined using a multiplex immunoassay kit (U-PLEX Mouse diabetes assay, Bio-Plex Pro, Bio-Rad, Belgium) and measured using Luminex technology (Bioplex, Bio-Rad, Belgium) according to manufacturer’s instructions.

### Histological analyses

Subcutaneous adipose tissue depots were fixed in 4% paraformaldehyde for 24 h at room temperature. Samples were then immersed in ethanol 100% before processing for paraffin embedding. To determine the adipocyte diameter, paraffin sections of 5 µm were stained with hematoxylin and eosin. Images were obtained using a SCN400 slide scanner and digital Image Hub software 561 (Leica Biosystems, Wetzlar, Germany). Adipocyte diameter was determined using ImageJ (National institutes of health, Bethesda, MD, USA). F4/80 positive areas in the adipose tissue were randomly counted after immunostaining with F4/80 antibody (Ab6640, Abcam, Cambridge, UK). All histological observations were analyzed in a blinded manner by three individuals. At least 5 high-magnification fields/mice were randomly selected and obtained using SCN400 slide scanner and digital image hub software (Leica Biosystems, Wetzlar, Germany).

Analysis of the mucus layer and goblet cells was made as previously described.^89^ Briefly, paraffin sections of 5 μm were stained with alcian blue. Images were captured at ×20 magnification and obtained using a SNC400 slide scanner and digital Image Hub software 561 (Leica Biosystems, Wetzlar, Germany). Analyses were performed using ImageJ (version 1.48 r, National Institutes of Health, Bethesda, Maryland, USA) in a blinded manner. For the mucus layer thickness, two to four fields were used for each mouse and a minimum of 50 different measurements (up to 200) were made perpendicular to the inner mucus layer per field. Each value represents the mean of the different measurements per field. For the goblet cells, the luminal side, muscularis mucosae, submucosa, and muscle layer were removed using ImageJ software and the blue area and the total area were measured separately in the remaining mucosal part of the colon. The proportion of the goblet cells was quantified based on the ratio between the blue area over the total area.

### RNA preparation and real-time qPCR analysis

Total RNA was prepared from tissues using TriPure reagent (Roche, Basel, Switzerland). Quantification and integrity analysis of total RNA were performed by analyzing 1 μL of each sample in an Agilent 2100 Bioanalyzer (Agilent RNA 6000 Nano Kit, Agilent, Santa Clara, CA, USA). cDNA was prepared by reverse transcription of 1 μg total RNA using a reverse transcription system kit (Promega, Madison, WI, USA). Real-time PCR was performed with the CFX Manager 3.1 software (Bio-Rad, Hercules, CA) using Mesa Fast qPCR (GoTaq qPCR Master Mix, Promega, Madison, WI, USA) for detection, according to the manufacturer’s instructions. *Rpl19* was chosen as the housekeeping gene. All samples were performed in duplicate, and data were analyzed according to the 2− ΔΔCT method. The quality of the amplified product was assessed by melting curve analysis at the end of amplification. The primer sequences for the targeted mouse genes are presented in Supplemental Table S2.

### Gut microbiota sequencing

Feces were sampled for gut microbiota analysis at the end of experiment. Genomic DNA was extracted using a QIAamp DNA Stool Mini Kit (Qiagen, Hilden, Germany), according to the manufacturer’s instructions, including a bead-beating step.

Cecal content was then used for sequencing analysis. Genomic DNA extracted from fecal content of the mice was diluted at 20 ng/µL and used as template for the amplification of the V4 region of the bacterial 16S rRNA gene with the primers 515 F (GTGYCAGCMGCCGCGGTAA) and 806 R (GGACTACNVGGGTWTCTAAT) using 30 amplification cycles with an annealing temperature of 65°C. High-throughput sequencing of purified amplicons was analyzed using Illumina MiSeq cartridge, according to the manufacturer instructions. As MiSeq sequencing enables paired 300-bp reads, the ends of each read overlap and can be stitched together to generate extremely high-quality, full-length reads covering the entire V4 region. The quality of the run was checked internally using PhiX, and for further analysis, each pair-end sequence was assigned to its sample using the previously integrated index. The resulting reads were processed through FROGS pipeline implemented on a Galaxy instance.^90^ The sequences were de-replicated and clustered using swarm method with an aggregation distance equal to 3 for the clustering.^91^ Chimeras were removed using the Vsearch tool.^91^ Sequences were then filtered to keep clusters, also called operational taxonomic units (OTUs) present in at least 7 samples and representing 0.00005% of all sequences. The taxonomic affiliations were performed using 16S SILVA database (Silva SSU_138, Max Planck institute, Bremen, Germany). The average number of sequences per sample was 695,950 sequences. Alpha diversity (Simpson and Shannon) and the Jaccard-Binary metric were performed using FROGS. A metric multidimensional scaling (MDS) plot, using the Jaccard-Binary metric, was created with R. Multivariate ANOVA statistical analysis with Adonis was performed to determine the statistical differences of microbial community among the different diet groups. This test used the Jaccard-Binary dissimilarly matrix as the input and was performed over 9999 permutations. The resulting p-value is the result from diet comparison at the end of the experiment. Abundance of phyla and genera was calculated as percent abundance of OTUs present in the entire microbiota.

### Bile acid and short-chain fatty acid quantification

BA and SCFA were quantified using HPLC-MS methods, as previously described.^92^ Briefly, for BA analysis, plasma samples were placed in acetone (in the presence of seven deuterated internal standards) to allow for protein precipitation. Next, samples were centrifuged, supernatants were recovered and evaporated under a stream of nitrogen. Organic residues were then resuspended in methanol. Samples were analyzed using an LTQ-Orbitrap XL using an electrospray probe in negative mode. Chromatographic separation was achieved using an Ascentis Express C-18 column (4.6 × 100 mm, 2.7 µm) (Sigma-Aldrich) and a gradient of water and acetonitrile in the presence of formic acid. For SCFA analysis, the cecal content (50–60 mg wet material) was homogenized in water followed by sonication in an ice water bath. Acetonitrile was used for protein precipitation (in the presence of valproic acid as internal standard). Following centrifugation, the supernatant was recovered and a derivatization step (using 3-nitrophenylhydrazine in the presence of EDC and pyridine) performed. Samples were purified using liquid-liquid extraction to remove the remaining reagents. After evaporation, the final residue was analyzed using an LTQ Orbitrap XL mass spectrometer coupled to an Accela HPLC system (ThermoFisher Scientific). A Hypersil GOLD PFP (100 × 2.1 mm; 1.9 μm) column using a gradient of water-acetonitrile-acetic acid and acetonitrile-acetic acid allowed separating the different isomers. For ionization, an APCI probe was used in positive mode. For each cecal content, an aliquot was freeze-dried to determine a dry residue that was used for data normalization. For both types of analytes, calibration curves were prepared using the same conditions to determine sample content. Xcalibur® software was used for data analysis.

### Statistical analysis

Mouse data are expressed as the mean ± S.E.M (standard error of mean). Statistical analyses were performed using Graphpad Prism for Windows (version 9.00; graphpad software, San Diego, CA, USA). One-way or two-way analysis of variance (ANOVA) was performed, followed by appropriate post-hoc tests (Dunnett’s or Bonferroni, respectively) when differences were significant. Bacterial DNA sequencing was analyzed using Kruskal–Wallis test with Dunn’s multiple comparison test. A p-value < 0.05 was considered significant. For all analyses, exclusion decisions were supported by the Grubbs test for outlier detection.

## Supplementary Material

Supplemental Material

## Data Availability

All data generated or analyzed during this study are included in this published article and its supplementary information files. The raw amplicon sequencing data analyzed in this study will be accessible in the European Nucleotide Archive (ENA) at EMBL-EBI under accession number https://www.ebi.ac.uk/ena/browser/view/PRJEB56494.

## Authors’ information

^a^ Metabolism and Nutrition Research Group (MNUT), Louvain Drug Research Institute (LDRI), UCLouvain, Université catholique de Louvain, Av. E. Mounier, 73 B1.73.11, 1200 Brussels, Belgium, ^b^ WEL Research Institute (WELRI), Walloon Excellence in Life Sciences and BIOtechnology (WELBIO), avenue Pasteur, 6, 1300 Wavre, Belgium, ^c^ Bioanalysis and Pharmacology of Bioactive Lipids Research Group (BPBL), Louvain Drug Research Institute (LDRI), UCLouvain, Université catholique de Louvain, Av. E. Mounier, 72 B1.72.01, 1200 Brussels Brussels, Belgium, ^d^ current address: Institute of Biomedicine, Department of Medical Biochemistry and Cell Biology, University of Gothenburg, Gothenburg, Sweden
