# Supplementary figures and images for: Inulin increases the beneficial effects of rhubarb supplementation on high-fat high-sugar diet-induced metabolic disorders in mice: impact on energy expenditure, brown adipose tissue activity, and microbiota
Source: Gut Microbes. 2023 Feb 20;15(1):2178796. doi: 10.1080/19490976.2023.2178796 (PMC9980659; doi:10.1080/19490976.2023.2178796)

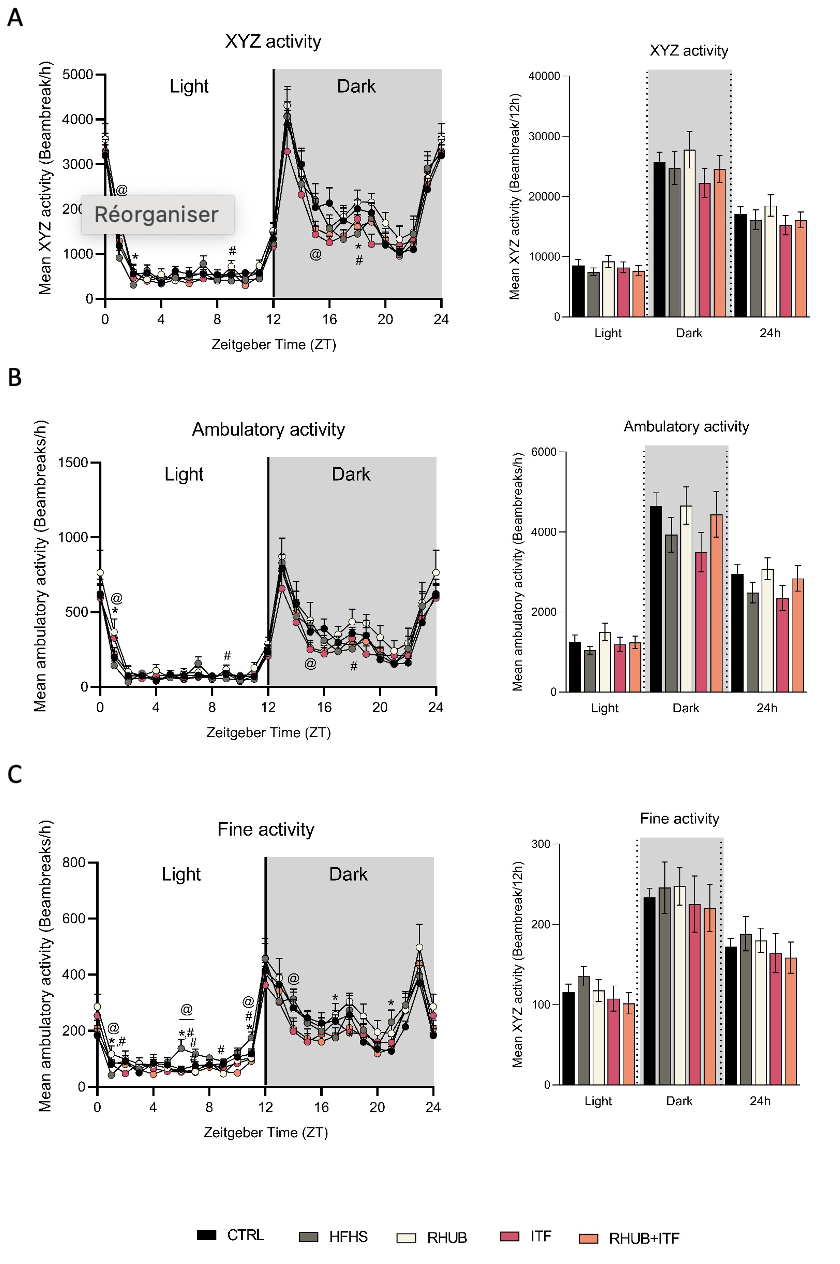

Supplement: Supplemental Material [file KGMI_A_2178796_SM7937.zip › 10_Supplementary_Fig_1.tiff]

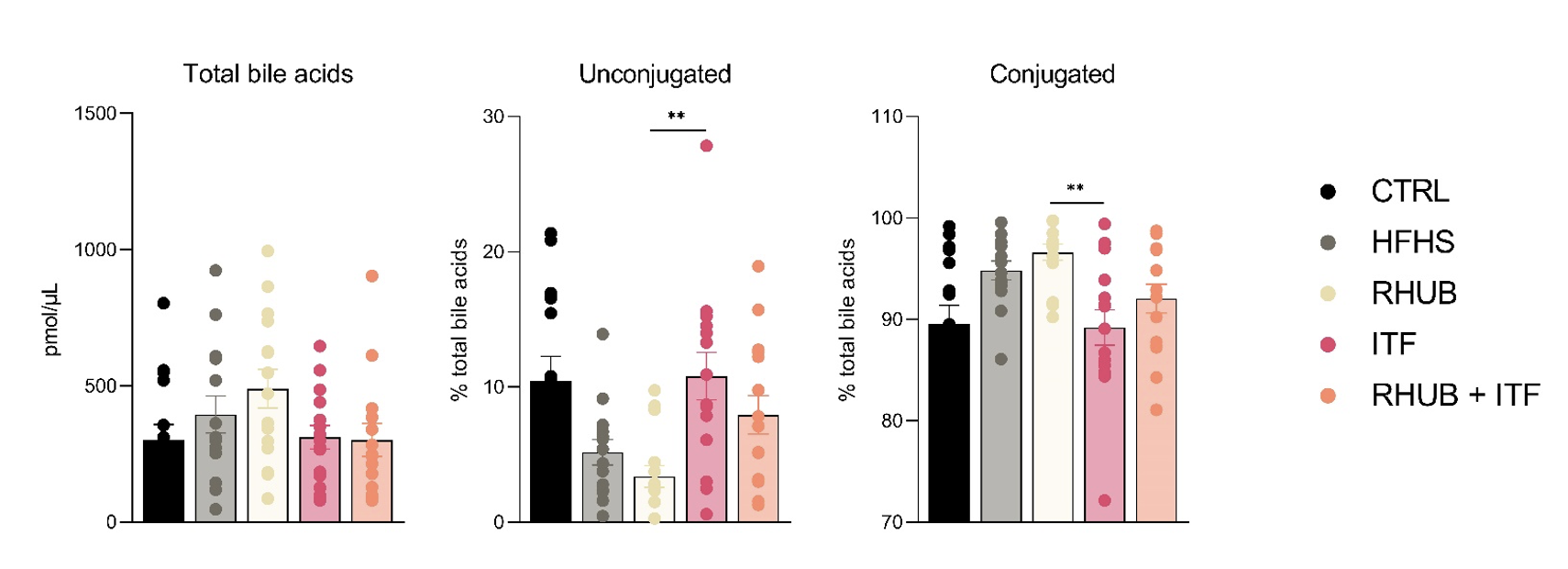

Supplement: Supplemental Material [file KGMI_A_2178796_SM7937.zip › 11_Supplementary_Fig_2.tiff]
